# The impact of medical tourism and the code of medical ethics on advertisement in Nigeria

**DOI:** 10.11604/pamj.2014.19.103.5217

**Published:** 2014-09-29

**Authors:** Olusesan Ayodeji Makinde, Brandon Brown, Olalekan Olaleye

**Affiliations:** 1 Measure Evaluation/JSI, 90 Nelson Mandela Street, Asokoro, Abuja, Nigeria; 2 Department of Population Health and Disease Prevention, University of California, Irvine, USA; 3 Department of Obstetrics and Gynaecology, Lagoon Hospital Ikeja, Lagos, Nigeria

**Keywords:** Advertising, consumer health information, health facilities, health policy, healthcare provider, facility regulation, medical ethics, medical tourism, nigeria, physicians

## Abstract

Advances in management of clinical conditions are being made in several resource poor countries including Nigeria. Yet, the code of medical ethics which bars physician and health practices from advertising the kind of services they render deters these practices. This is worsened by the incursion of medical tourism facilitators (MTF) who continue to market healthcare services across countries over the internet and social media thereby raising ethical questions. A significant review of the advertisement ban in the code of ethics is long overdue. Limited knowledge about advances in medical practice among physicians and the populace, the growing medical tourism industry and its attendant effects, and the possibility of driving brain gain provide evidence to repeal the code. Ethical issues, resistance to change and elitist ideas are mitigating factors working in the opposite direction. The repeal of the code of medical ethics against advertising will undoubtedly favor health facilities in the country that currently cannot advertise the kind of services they render. A repeal or review of this code of medical ethics is necessary with properly laid down guidelines on how advertisements can be and cannot be done.

## Introduction

Despite prevalent political and economic challenges, medical practice in Nigeria has experienced remarkable progress in recent years. Modern day management centers are increasingly abundant, with the capacity to carry out advanced medical procedures and refinement of common procedures (advanced fertility treatments, minimal access surgeries, transplant procedures, joint replacement surgeries and availability of improved diagnostic techniques, etc). Many of these advances in the management of patients are due to significant investments in infrastructure and training. Knowledge of the advances being made especially in private health facilities are limited to those working within their immediate environment. Many physicians practicing in different parts of the country do not know of the advances in health service delivery now available in the country, less so, the ill patient who may require these services as an emergency or lifesaving intervention. The poor level of awareness and knowledge of available services by physicians and the general population can be attributed to a long standing medical code which bars orthodox health practitioners from advertising their services [1].

In addition, there is a scarcity of scientific conferences and interdisciplinary seminars to foster the dissemination of useful clinical and research knowledge among stakeholders. The Code of Medical Ethics in Nigeria: Rules of Professional Conduct for Medical and Dental Practitioners in Part F (Self advertisement and related offences: relationship with the media) particularly frowns at public advertisements by physicians and health facilities and only permits the distribution of patient information leaflets on services offered to patients and their relatives within the premises of the health facilities [1]. Similar codes were in effect in several developed countries prior to their repeal [2, 3]. To further worsen the precarious situation is the growing incursion of unorthodox physicians within the country – who are not bound by similar laws – and the rise in international medical tourism [4, 5], coupled with the aggressiveness being undertaken by these foreign institutions and their marketers to attract clients from other countries. Thus, it has become imperative to voice concerns on the danger that the persistence of this medical code poses to the Nigerian public as well as the medical, ethical and socio-economic impact on both patients and hospital owners.

Medical tourism, which is the travel to another country for the purpose of treatment, is growing. The increasing recruitment of patients from Nigeria provide a call to action for hospital owners, the Association of General and Private Medical Practitioners in Nigeria (AGPMPN), the Nigeria Medical Association (NMA) the Federal Ministry of Health and other stakeholders to begin advocacy for the repeal of the code of medical ethics against advertisement by physicians and health practices by the Medical and Dental Council of Nigeria (MDCN). As a result of the growth, a new terminology and accompanying industry “Medical Travel Facilitators” or “Medical Tourism Facilitators” (MTF) has emerged comprising of individuals and companies that connect patients to medical providers across countries [6, 7]. The growth in this industry has been fostered by rising cost in healthcare and lengthy wait times in developed countries [5, 8], limited number of specialist practitioners, poor information on services within countries, and distrust in the quality of care by local healthcare providers in developing countries [5, 6]. Medical travel from the developing world used to be mainly by the affluent in these countries.

However, the increasing availability of world class health facilities in low and middle income countries (LMIC) and the expanding middle class in several developing countries who can pay for these services, has increased the population traveling to other countries for care. India is one of these destination countries. They have facilitated potential economic gains in this industry with the creation of a class of visa for medical tourism, and with projected economic growth to reach US$ 69 Billion (8.5% of the GDP) in the next few years [9]. Some of the destination countries governments’ have favorably supported the growth of this economically viable sector [5, 10, 11]. Such government's support of private healthcare businesses is obviously lacking in departure countries like Nigeria, and the tide must be turned around to ensure these governments can offer their citizens the best within their country which is the essence of governance.

The increased access to internet services via mobile telecommunication service providers in Nigeria, in addition to the rapidly growing subscriber base of social media communities like Facebook, Twitter, LinkedIn and Instagram have created viable marketing channels for cross border advertisement. This has facilitated the recruitment of patients at the detriment of health facilities in the country which continue to experience underutilization of their facilities [12]. The modalities these external institutions use to reach out to patients are quite innovative and continue to cause significant imbalance for health facilities within the country that do not have the opportunity to advertise the kind of services they render. Additionally, the ethical implications that these cross border health marketing results in are diverse which are often significant undertones hardly considered by the medical tourists.

This paper argues for the repeal or significant review of the code of medical ethics which bars healthcare providers and institutions from advertising the kind of services rendered at their medical practices.

### Factors impacting service advertisement

Healthcare providers invest in equipment and acquire knowledge and skills that will help provide better quality and up-to-date services to the population they serve while at the same time sustain their practices. While this can include significant strategic investments, most physicians and practices do not adequately reap the benefits of the effort and investments. Part of this gap in fulfilling their potential may be attributable to their inability to advertise the kind of services they offer at their health facilities.

About 30, 000 Nigerians are estimated to spend about US$1 Billion annually on medical tourism [13]. Should these resources be committed to the local health market, it would boost local industry. At a public lecture organized by the Kelina Hospital Abuja and the Surgical Aid Foundation in Abuja, in March, 2014, the hospital presented their efforts at improving the modalities of management of nephrolithiasis (renal stones) with the launch of hospital equipment aimed at delivering minimally invasive procedures to the teeming population that have this condition in the country. Whilst they have made this investment, they lamented the difficulty in raising awareness about the sophisticated and modern day management services they could offer to the Nigerian population that need such services. Kelina hospital took the pain to invite a renowned Indian surgeon from one of the popular medical tourist destination hospitals to come observe procedures carried out at the health facility during a weeklong medical mission. Feedback from the Indian specialist was to help address concerns about the quality of services rendered. This is just a single case among the several others that abound along the corridors of the country. The effect of the medical code barring health practices from advertising in the country is far reaching beyond the shores of the country and does not promote an even playing field for health facilities. The impact of this restriction affects negatively the ability of health facilities in-country to stay viable and competitive in the market. A repeal or significant review of this code of medical ethics is necessary as the metamorphosis in the medical industry has changed the platform for doing medical businesses. The promoting factors toward the repeal and the mitigating factors against the repeal of the code of medical ethics on advertisement are further discussed below.

### Promoting factors Knowledge about advances in clinical management is limited

Though several advances are being made in clinical practice within the country, few patients are able to seek and access them because of the limited information on where these services are offered even when they have the financial resources to pay for the services. Such limited information negatively impacts the choices of management by physicians. In addition, this equally has a negative effect on the practices providing specialized care and the profit of the health facility. Should there be an increase in the utilization of these services, the chances that the fees for these specialized services will be reduced and better affordable by patients in the country is high. It is noteworthy that 33% of health facilities in Nigeria are private health facilities with majority of their clientele base paying for services out-of-pocket [14, 15]. The more affordable the services, the more likely that people will access them and the more the physician will have an opportunity to improve his/her clinical acumen.

### Effect of medical tourism

Medical tourism is increasingly popular for Nigerians as a result of a wide range of factors. The affluent population perceive as an elitist idea, travelling abroad for medical routine physical examinations and surgically amenable ailments. In addition, the perceived suboptimal quality of care and treatment options available at healthcare facilities in the country have resulted in low patronage by those that can afford the relatively expensive healthcare cost in more developed countries. This costs the health industry significantly in lost revenue, and also the country the cost of paying for these services when these patrons are government officials thereby negatively impacting the Nigerian health market. Many argue that the quality of service in the country may not be comparable to the countries that receive the patients. However, few may be aware that a Joint Commission International (JCI) accredited health facility is available within the country. JCI is the “International leader in Healthcare Accreditation around the world and is the oldest and largest standards setting and accrediting body in health care in the United States” [16]. JCI is the main accreditation organization which has accredited many of the highly sought after health facilities in the destination countries that medical tourists patronize [4, 5]. The medical code against advertising by in-country hospitals and physicians places them at a disadvantage since others from outside the country are not bound by similar laws. Should the profession across the globe have been kept sacrosanct and free of advertisements, there may not have been need for this debate for repeal because of an uneven advantage. Though strong opposition to advertising by physicians exists and is still maintained as a law in several countries including some developed countries [17], the uneven playing field calls for a rethink of this code especially in developing countries that have a growing middle class that can pay for services. The internet has opened up a whole new global village which makes enforcing such advertisement laws in a country difficult and polarized. Such, if allowed to continue will continue to result in inequity of knowledge and access to the population in a country.

Patients travelling out of the country for the purpose of medical treatment who do so because they are unaware of the opportunities for similar management within their country are being done a disservice. Most times, they utilize the internet to scout for services which promptly meets them with a wide range of MTFs and health facilities across the globe with several opportunities for care. The cost of travelling for care most times with at least a companion if channeled into the management of the patient in-country would cover the cost of treating the patient with significant left over. Additionally, the cultural value of supportive management by family members – very popular in our environment – is eroded since only a few can accompany the patient to the location for his/ her care. It is difficult to tell exactly how much of psychological effect the absence of this family support can lead to. One also cannot rule out the negative eventual outcome of a loss of life while on a medical trip abroad. This increases the cost of management exponentially as fees for transporting the remains of a family member back to the country can be high.

Furthermore, patients travelling for medical tourism will hardly research on the laws governing medical practice in the countries they visit and will probably assume similar laws in their countries are in place. Such assumption can be quite wrong and misleading in cases that result in litigation.

### Can drive brain gain?

Many resource poor countries including Nigeria have suffered brain drain over the years, with the brightest scientists becoming specialists in different countries. Turning brain drain into brain gain requires that the specialists be properly engaged to remain in Nigeria and provide such specialized care which they have garnered over the years. This will be possible when they can properly advertise what they bring to the country and the period and location for which they can be contacted while in-country. Should the visiting physician see as rewarding a period in his home country, the chance that he/ she will continue such visits is high. During such visits, there can also be a transfer of knowledge and skills to those in the country who can continue to provide these services to the population.

### Mitigating factors

#### Ethical issues

Ethics remain the greatest barrier to the repeal of this code. Tomycz in his 2006 paper states that “the best advertisement for doctors is not advertising”. His opinion that the physician profession is sacred and as such should not be communized with other professions is questionable [18]. Several countries including Nigeria continue to prevent advertisement by physicians and health practices as it is believed that the physician interest will override that of the patient if advertisements were permitted. However, until lately, no one had looked carefully at the effect of advertising bans on the practice of medicine. Zwier in her paper outlines the other side of the debate. Advertising can be an opportunity to let the public know the variation in the modalities of care for a particular condition [17]. It can help enlighten the populace more about treatment options rather than leave the decision absolutely to the Physician. Cases of Physician-Induced-Demand can be reduced if patients are better informed about treatment options. Patients will rather go through a process that will have the shortest recovery time and fewer possible complications like medical management or minimal access surgery for renal stone removal than undergo open abdominal surgery for the same condition [19]. In event that the attending physician is only skilled in open abdomen surgery, the likelihood that he will lay the option of minimal access surgery to the patient are slim even when the patient can afford these services and they are available within reach. The growth of the internet and medical tourism further brings to bear a different ethical perspective. As the world is now a global village, there is an increasing intrusion of international health adverts and this also is ethically wrong if the same rules which operate in a country cannot be applied to these health institutions. One can reasonably argue that should the MDCN and other stakeholders fail to repeal or modify significantly the law barring physicians and health practices from advertising, then they must protect the interest of health providers in the country and apply similar laws to international patient recruiters and health institutions from targeting the Nigerian market with their adverts.

#### Resistance to change

Resistance to change is a very popular mental model in organizational development and psychology. People resist change because of the fear of the unknown and will rather maintain the status quo than explore something adventurous with an unknown but possibly better outcome. Certainly, exploring advertising by physicians and health practices requires a well thought out and well governed process to ensure that this opportunity is not abused and unduly taken advantage of. Countries that have opened up physician practices to advertisements have put in place guidelines that govern this area and such models should be embraced should the MDCN repeal or modify the code governing advertisement by physicians in Nigeria [20]. Oreg in his 2003 paper identified 6 sources of resistance related to an individuals’ personality and these are: i) reluctance to lose control ii) Cognitive rigidity iii) Lack of psychological resilience iv)intolerance to the adjusted period involved in change v)preference for low levels of stimulation and novelty, and vi) reluctance to give up old habits [21]. These are diverse opinions which are directly applicable to the Nigerian health industry and its stand on advertisement.

#### Elitist idea

It is becoming increasingly apparent that Nigeria has a segregated social system where socioeconomic status has an effect on the choice and utilization of health services [22]. The Demographic and Health Surveys have consistently highlighted the significant divide in health status by different social classes in the Nigerian society. These are points of concern as it shows the inequity to access and utilization of modern health care services in our environment. Such differences will not abate if access to specialist services remains available only to the elite and few that can afford to find a foreign institution offering a service.

## Conclusion

The continued restriction of in-country health facilities and physicians from advertising their services is unduly harsh and does not provide an equal trade zone for health facilities and the entrepreneurs who invest in them. Whilst advertising by health facilities and physician practices can be potentially gainful, there are several potential ethical implications. Though this argument favors a repeal or significant review of the medical code against advertising by Physicians and health practices for a more flexible one that does not put them at a disadvantage, proper governance and guidelines are required prior to its implementation. Medical tourists are concerned about quality of care, and a transparent process which demonstrates quality in health service delivery by in-country health facilities is needed.
